# Demonstrating the robustness of population surveillance data: implications of error rates on demographic and mortality estimates

**DOI:** 10.1186/1471-2288-8-13

**Published:** 2008-03-25

**Authors:** Edward Fottrell, Peter Byass, Yemane Berhane

**Affiliations:** 1 Umeå International School of Public Health, Epidemiology and Public Health Sciences, Umeå University, Sweden; 2 Addis Continental Institute of Public Health, P.O. Box: 26751/1000, Addis Ababa, Ethiopia

## Abstract

**Background:**

As in any measurement process, a certain amount of error may be expected in routine population surveillance operations such as those in demographic surveillance sites (DSSs). Vital events are likely to be missed and errors made no matter what method of data capture is used or what quality control procedures are in place. The extent to which random errors in large, longitudinal datasets affect overall health and demographic profiles has important implications for the role of DSSs as platforms for public health research and clinical trials. Such knowledge is also of particular importance if the outputs of DSSs are to be extrapolated and aggregated with realistic margins of error and validity.

**Methods:**

This study uses the first 10-year dataset from the Butajira Rural Health Project (BRHP) DSS, Ethiopia, covering approximately 336,000 person-years of data. Simple programmes were written to introduce random errors and omissions into new versions of the definitive 10-year Butajira dataset. Key parameters of sex, age, death, literacy and roof material (an indicator of poverty) were selected for the introduction of errors based on their obvious importance in demographic and health surveillance and their established significant associations with mortality.

Defining the original 10-year dataset as the 'gold standard' for the purposes of this investigation, population, age and sex compositions and Poisson regression models of mortality rate ratios were compared between each of the intentionally erroneous datasets and the original 'gold standard' 10-year data.

**Results:**

The composition of the Butajira population was well represented despite introducing random errors, and differences between population pyramids based on the derived datasets were subtle. Regression analyses of well-established mortality risk factors were largely unaffected even by relatively high levels of random errors in the data.

**Conclusion:**

The low sensitivity of parameter estimates and regression analyses to significant amounts of randomly introduced errors indicates a high level of robustness of the dataset. This apparent inertia of population parameter estimates to simulated errors is largely due to the size of the dataset. Tolerable margins of random error in DSS data may exceed 20%. While this is not an argument in favour of poor quality data, reducing the time and valuable resources spent on detecting and correcting random errors in routine DSS operations may be justifiable as the returns from such procedures diminish with increasing overall accuracy. The money and effort currently spent on endlessly correcting DSS datasets would perhaps be better spent on increasing the surveillance population size and geographic spread of DSSs and analysing and disseminating research findings.

## Background

The majority of people living in the world's poorest countries, where the burden of disease is highest, remain outside of any kind of systematic health surveillance [1-6]. Without appropriate population-based data it is not possible to reliably document the health status of populations or the progression of epidemiological transitions, and there is virtually no capacity to evaluate interventions. This can result in inappropriate or misplaced interventions and direct valuable resources away from relevant and effective health programmes.

Sample-based mortality surveillance addresses some of these issues and is a useful method for monitoring trends over time and differentials between sub-groups [7]. Based on this, a number of Demographic Surveillance Sites (DSSs) have been established in developing countries over the past two decades. A DSS is a set of field and computing operations to handle the longitudinal follow-up of well-defined entities or primary subjects (individuals, families, and residential units) and all related demographic and health outcomes within a clearly circumscribed geographic area [4,6].

As in any measurement process, a certain amount of error is to be expected in DSS data. Measurement errors may occur in a variety of ways: instrumental errors arising from imprecise instruments or questionnaire limitations; underlying variability leading to differences between replicate measurements taken at different time points; respondent errors arising through misunderstanding, faulty recall or reporting bias; observer error, including imprecision and mistakes; and data processing errors during coding and data entry [8]. These errors can be categorised into two groups: namely, systematic or random errors, which are defined in the present paper as follows.

Systematic errors relate to the study design, methods and tools utilised and may be more common in certain demographic groups or regions. For example, systematically missing the deaths in a specific age or sex group, such as infants, due to excessively long periods between surveillance rounds combined with cultural reporting biases introduces systematic errors. Excessive differential bias (i.e. bias that does not affect everyone equally) is unacceptable if realistic and useful estimates of infant mortality rates are to be made. It is essential, therefore, that the design and routine operations of surveillance activities consider all possible sources of error, attempt to detect such errors and implement strategies to prevent or correct shortcomings. It is best to pre-empt systematic errors, as correcting them after data have been collected is often extremely difficult and resource intensive. In practice this demands thorough field testing and careful design of survey tools and methodologies.

Random errors occur independently of study design and methods used, and are unrelated to the value of other variables in the dataset. Divergence of an observation, due to chance alone, leads to a lack of precision in the measurement of an association. Key sources of random errors in large databases are mismeasurement and mistakes in data recording. For example, mistakes in recording data or entering it into the database, such as transposition of numbers, are random errors if they are unrelated to the particular variable of interest and other characteristics of that particular case. The accumulation of random errors in longitudinal surveillance is potentially a major problem that may invalidate the results of otherwise well-designed studies. The effects of random error are reduced with increasing sample size [9].

Measurement error often has some systematic and some random components [9]. The problems that may result from errors that occur when measuring exposure or outcome variables generally relate to false population representation and univariate regression dilution bias, whereby estimated regression coefficients of single exposure-effect estimates may be biased towards the null value of no exposure effect, so that the magnitude of the association between the exposure and the outcome will tend to be underestimated – the association is said to be attenuated [8-10]. The effect of random error in multivariate analyses, with errors in a number of interrelated variables, can lead to more complex, serious distortions in the estimation of real exposure-outcome associations [11,12]. All types of non-differential random measurement error reduce the chance that true significant associations will be identified; in other words they reduce statistical power [9].

To draw correct quantitative conclusions that can form the basis for public health intervention necessitates that the effects of measurement error are appreciated and accounted for [11]. Sensitivity of data to error, often termed 'robustness', is important in assessing the degree of uncertainty associated with surveillance outputs [10]. Various methods are available to correct measurement errors, the aim being to simulate true population profiles and exposure-outcome effects which would be observed if errors were eliminated [12-16]. In epidemiology and population measurements, however, a truly gold standard measure can rarely be used for validation studies, and the use of imperfect or 'alloyed' gold standards risks introducing more bias than they are correcting [17]. As such, correction techniques are seldom used in epidemiological studies [18] and it is difficult to regain lost power using statistical fixes [9].

For these reasons a significant amount of DSS operations and resources are dedicated to data quality assurance mechanisms. Checking completed surveillance tools for evident errors and omissions is a crucial aspect of this and is often performed at every level of field organisation with checks becoming more detailed as data progress through the system [6]. Questionnaires with obvious errors or omissions that cannot be corrected by supervisors are returned to the field while those that are free of errors proceed to data entry, which in some settings is performed twice to enhance data quality. Random duplicate household visits are often considered to be an additional important aspect of data quality assurance. Of the 37 member sites of the Indepth network (International Network of field sites with continuous Demographic Evaluation of Populations and Their Health), 19 describe scheduled random re-visits of primary sampling units as a method of data quality control, with the percentage of households re-visited ranging from 2% (Agincourt DSS, South Africa) to between 5 and 10% (Nouna DSS, Burkina Faso). Several DSS sites that perform re-visits do not specify the percentage of the total population revisited [6,19].

There is surprisingly little literature relating to data quality and error rates in DSSs. In their assessment of a computerised approach to the management of an epidemiological field trial in Farafenni DSS, Gambia, Rowan et al. (1987) identified 383 errors in 13 fields of a weekly morbidity surveillance questionnaire conducted over 18 weeks, giving an overall error rate of 0.29%. Almost three quarters (71.8%) of these errors (n = 275) were correctable by staff in the field office without having to refer back to the field. A further 28.2% of the errors were correctable on referral back to the field. Thus a total 96.3% of the errors were correctable, giving a final detectable error rate of 0.01% [20].

Despite the best efforts of DSS operations, it is unlikely that measurement error can be completely eliminated. It is therefore important to have some appreciation of the implications of measurement error for DSS study results and tolerable levels of errors and missing data. This paper attempts to develop understanding of the effects of measurement error on the results and conclusions drawn from DSS data, with particular emphasis on mortality measurements. In doing so, it is anticipated that the sensitivity of DSS data to random errors and omissions can be determined. Such knowledge is of particular importance if the outputs of population surveillance, or indeed other large community-based surveys in developing countries, are to be extrapolated and aggregated with realistic margins of error and validity. This work also addresses the widespread need for refined and evidence-based procedures in demographic and health surveillance where endless re-checking and multiple re-visits to households is an expensive and time-consuming pursuit.

## Methods

The Butajira Rural Health Programme (BRHP) in Ethiopia has maintained a programme of epidemiological surveillance in the Butajira district, some 130 kilometres south of the capital, Addis Ababa, since 1987. The basic operations of BRHP are typical of DSS systems and of the member sites of the Indepth network [19]. Continuous community-based surveillance of an open cohort population sample is conducted through household surveys relating to births, deaths and migrations, as well as socioeconomics and living conditions. Given its wealth of data, BRHP acts as a platform for more specific epidemiological and public health studies. This study uses BRHP data collected between 1^st ^January 1987 and 31^st ^December 1996. This 10-year dataset covers approximately 336,000 person-years and has been used extensively to describe the population of Butajira and patterns of morbidity and mortality in the district [4].

Simple programmes were written using Microsoft Visual FoxPro software to simulate versions of the 10-year Butajira dataset containing random errors and omissions as outlined in Table 1. Key parameters were selected for the introduction of errors based on their obvious importance in demographic and health surveillance and their established significant associations with mortality in the Butajira setting. A random selection of 10% or 20% of cases was randomly assigned data values for sex (male or female). Age in years is determined for each individual in the dataset from the recorded date of birth and in a randomly selected 10% of the cases this calculated age value was increased by 10 or 20% of its 'true' value. Whether an individual in the surveillance population died during the 10-year period is recorded in the dataset, and versions of the dataset were generated whereby a randomly selected 10 or 20% of cases recorded as having died had information about their death removed, thereby simulating missed events. Literacy and the material used to construct one's roof are important indicators of socioeconomic status, which has well-established associations with mortality in the Butajira setting. Therefore simulated versions of the gold standard dataset were created in which information on whether an individual was literate was removed in a randomly selected 10% of cases and the values 'corrugated' or 'thatched' were randomly assigned to a random selection of 10% of the cases.

**Table 1:** Overview of versions of the BRHP 10-year data showing parameters used, simulated errors and outcomes analysed.

	Random Errors Introduced					
	Age	Sex	Deaths	Literacy data	Household Roof material	Analysis

'Gold Standard'	No errors	No errors	No errors	No errors	No errors	

1	10% increase in 10% of cases					Population pyramids and mortality rate ratios
2		10% randomised in 10% of cases				
3	10% increase in 10% of cases	10% randomised in 10% of cases				
4	20% increase in 10% of cases	20% randomised in 10% of cases				

5			10% deaths randomly missed			Mortality rate ratios
6		10% randomised in 10% of cases	10% deaths randomly missed			
7				10% literacy randomly missing		
8					10% of roofs randomised	
9	10% increase in 10% of cases	10% randomised in 10% of cases	10% deaths randomly missed	10% literacy randomly missing	10% of roofs randomised	

There are no standard procedures for this type of investigation and therefore the parameter modification described above is arbitrary. Nevertheless, the extent of parameter modification in this study was influenced by probable random error margins in routine DSS procedures, which are unlikely to exceed 10% in most instances, as well as by issues of presenting the results – in our experiment, parameter modification of less than 10% failed to show any substantial differences in population representation and mortality patterns. In reality, random errors are unlikely to occur at a fixed rate and the introduction of set levels of error in this study was done to simplify the modelling process, with no attempt being made to represent systematic failures (which may have been possible by simulating age heaping or more random degrees of misreporting).

Population age and sex composition and all-cause mortality rates were calculated for each dataset and results were compared with the gold standard of the original 10-year data to determine the extent to which the introduction of errors affected the data's ability to represent the surveillance population. Rate ratios relative to other groups within a population are simple summary measures of populations that identify the most vulnerable groups within a population and are relevant to local users of surveillance data for the purposes of public health planning and priority setting. As such, multivariate Poisson regression models of mortality rate ratios for the 10-year dataset as well as each of the simulated error datasets were created to investigate the extent to which errors altered well-established associations between the above parameters and mortality.

## Results

**Figure 1:**
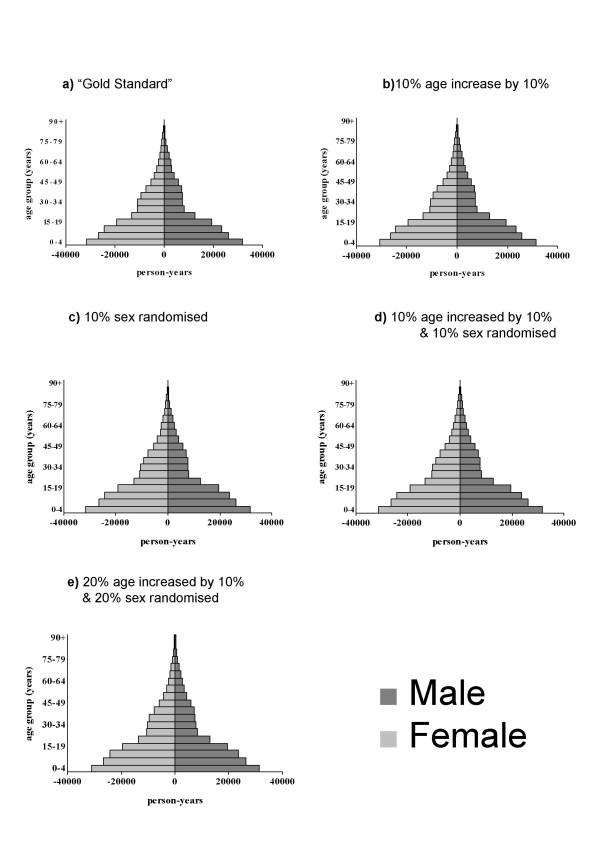
Population age and sex distributions for BRHP dataset with: a) no errors ('Gold Standard'; b) 10% of age data increased by 10%; c) 10% sex randomized; d) 10% age data increased by 10% and 10% sex data randomised; 20% age data increased by 10% and 20% of sex data randomized.

Figure 1 shows the population pyramids for Butajira based on the gold standard data (Figure 1a) as well as the erroneous datasets. A slight narrowing and increase in the height of the population pyramid can be observed when a proportion of age data are increased (Figure 1b). Very little change in population composition can be observed when the sex variable is randomised (Figure 1c) or when errors are introduced in both age and sex variables (Figure 1d). Even with the combination of errors in age and sex at the 20% level, the only clearly observable change in population composition is related to the rise in age (Figure 1e).

**Figure 2:**
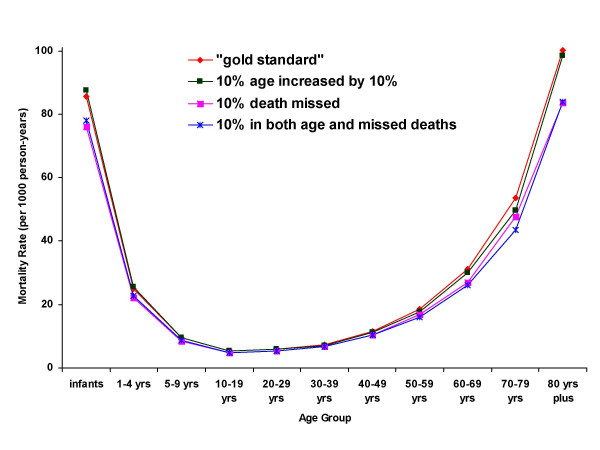
Age-specific mortality rates from 10-year BRHP data with and without simulated errors.

Figure 2 illustrates age-specific mortality rates based on the gold standard data and data containing errors in age and missed deaths. Random increases in age show little affect on the age-specific mortality profile. Missing death data have little noticeable affect on age-specific mortality rates between the ages of 5 and 60 years where substantial overlap of the lines representing the gold standard estimate and the erroneous estimates can be observed. At extremes of age, however, missing death information has a more noticeable effect and a widening of the gap between mortality rate estimates is noticeable.

Table 2 shows the univariate and multivariate Poisson regression models of mortality rate ratios for all versions of the dataset. All of the parameters show significant relationships with increased mortality in the gold-standard data and continue to do so when errors are introduced. Even when all errors are combined, the greatest change in the rate ratios in the univariate analysis is +0.14 for the thatched roof category and -0.14 for being in the oldest age group, however, neither of these changes affect the significant associations between the parameters and mortality.

**Table 2:** Multivariate Poisson regression models of mortality rate ratios, including different simulated errors, from 10-year BRHP data.

Parameter	Category	'Gold Standard'	10% of deaths randomly missed	10% of ages increased by 10%	10% of sex randomised	10% of literacy randomly missing	10% of roofs randomised	10% of all factors together
Age group:	20–49	Ref	Ref	Ref				Ref
	Under 20	2.17 (2.00–2.34)	2.14 (1.97–2.32)	2.21 (2.04–2.38)				2.18 (2.00–2.36)
	50-plus	3.99 (3.63–4.38)	3.90 (3.53–4.31)	3.94 (3.58–4.33)				3.85 (3.49–4.25)

Sex:	Female	Ref	Ref		Ref			Ref
	Male	1.18 (1.12–1.25)	1.17 (1.11–1.25)		1.17 (1.10–1.23)			1.16 (1.10–1.23)

Literacy:	Literate	Ref	Ref			Ref		Ref
	Illiterate	1.28 (1.21–1.36)	1.26 (1.19–1.34)			1.28 (1.21–1.36)		1.26 (1.18–1.33)

Roof:	Corrugated	Ref	Ref				Ref	Ref
	Thatched	1.75 (1.61–1.90)	1.78 (1.62–1.94)				1.59 (1.47–1.72)	1.61 (1.48–1.76)

Multivariate:	Under 20	2.20 (2.03–2.38)	2.17 (2.00–2.36)	2.25 (2.08–2.43)	2.20 (2.03–2.38)	2.20 (2.02–2.38)	2.19 (2.02–2.37)	2.20 (2.02–2.40)
	50-plus	4.05 (3.68–4.46)	3.97 (3.59–4.40)	4.01 (3.65–4.42)	4.06 (3.69–4.47)	4.00 (3.61–4.42)	4.03 (3.66–4.43)	3.86 (3.47–4.30)
	Male	1.15 (1.09–1.22)	1.14 (1.08–1.21)	1.15 (1.09–1.22)	1.14 (1.08–1.21)	1.16 (3.61–4.42)	1.15 (1.09–1.22)	1.15 (1.08–1.23)
	Illiterate	1.13 (1.07–1.20)	1.11 (1.04–1.18)	1.13 (1.07–1.20)	1.13 (1.07–1.20)	1.13 (1.06–1.20)	1.16 (1.09–1.23)	1.12 (1.05–1.20)
	Thatched	1.70 (1.56–1.86)	1.74 (1.59–1.91)	1.71 (1.57–1.86)	1.70 (1.56–1.86)	1.71 (1.56–1.88)	1.54 (1.43–1.67)	1.60 (1.45–1.75)

## Discussion

By assessing the implications of random errors and omissions on population descriptions and mortality estimates using surveillance data collected over a 10-year period in BRHP, this study has addressed important issues regarding the effects of measurement error on DSS study results. In an attempt to determine the effect of random errors, the original dataset was defined as a 'gold standard' which could be used for comparing population and mortality profiles when random errors were introduced into the data. It is important to emphasise that the original 10-year data is only described here as a gold standard for the purposes of these experiments and in reality it is inevitable that 10 years of accumulated demographic and epidemiological data will have acquired unknown random errors as well as possible systematic errors resulting from methods used in the routine surveillance operations in Butajira.

It is also important to emphasise that this work entirely separates random and systematic error and relates only to the implications of random error in the sense that it is assumed that any errors in measuring a variable are independent of the value of other variables. Whilst, in practical terms, missing 10% or 20% of deaths, for example, can be considered a systematic failure, the errors modelled in this study have been distributed randomly in the dataset – in other words, having missing or incorrect data is unrelated to any other factor in the dataset. In reality, some events are intrinsically more likely to be missed (e.g. very early deaths or deliberately concealed events) or misreported than others and this was not factored into this investigation. Such systematic errors are a superficial indicator of the quality of population data and are sensitive not only to the respondent's ability to recall their ages or dates of births accurately, but also to training procedures for enumerators, where staff may be explicitly discouraged from recording rounded ages. Modelling such errors may have diminished the generalisability and relevance of the findings of this study to surveillance operations overall, as they would need to relate to specific quality control procedures.

Population profiles from each version of the data represent Butajira's population composition well and differences between population pyramids based on datasets with age and sex errors are subtle. The results also show that 10% errors and omissions in six key parameters relating to mortality do not cause regression dilution bias to any large degree. Even when all errors are combined no major difference in rate ratios can be observed, although this may simply reflect little confounding between variables. Missed deaths have a more noticeable influence on age-specific mortality rates at the extremes of young and old age due to the fact that the majority of deaths in Butajira occur either within the first five years of life or during 'old age', and thus a 10% error involves a greater number in absolute terms. This implies that, in Butajira, accuracy of mortality measurement is perhaps more important in these age bands than in the 5 to 60 years age groups. This is likely to be due to the underlying disease characteristics of Butajira district, where HIV infection remains relatively low and mortality patterns reflect the resilience of communicable diseases as well as emerging non-communicable diseases (NCDs), consistent with features of delayed epidemiologic transition [21,22]. It is likely that in DSSs in settings with differing disease patterns, such as Agincourt DSS, South Africa, where the burden of HIV is large and where both males and females in the 20–34 age group are most at risk [23], or Purworejo DSS, Indonesia, characterised by a high burden of NCDs [24], differing concerns in relation to the detection and correction of errors for mortality estimates in certain age groups may apply. These priorities are likely to be influenced by localised issues of age-heaping, reporting bias and patterns of disease.

An important concept in all DSS operations is the point at which data are of sufficient quality to satisfy their intended purpose. Sentinel surveillance, population-based sample surveys and DSS activities in developing countries are a surrogate for more widespread, routine vital event surveillance. In settings where health budgets are small, complete vital event surveillance remains unaffordable and unrealistic. Nevertheless, it is these countries that need 'good' data so that scarce resources can be used appropriately. If the ultimate purpose of DSS operations, therefore, is to characterise the demographic and health profiles of localised populations in order to inform policy, when are 'good' data good enough for this purpose? The simulated errors described here had no affect on the results that would alter conclusions drawn from them, thus the policy implications in terms of identifying vulnerable groups and designing and targeting appropriate interventions should be negligible – perhaps this is a key characteristic of good data. Given that simulated error rates 1000 to 2000 times greater than that described by Rowan et al. (1987) had little effect on Butajira population and mortality profiles and mortality outcome-exposure associations, the benefits of attempting to eliminate relatively small error rates is questionable, not least with regard to how such efforts would affect conclusions and policy decisions based on the data [20].

Nevertheless, random and systematic errors and differential bias arising from specific methodologies, as well as sources of non-measurement bias, remain important concerns for surveillance systems. Continued efforts to detect such errors, investigation of their implications and strategies to prevent or correct shortcomings must continue to be given high priority in DSS operations. Furthermore, supervisory visits and data quality checking are important for providing constructive feedback to fieldworkers with the aim of improving interview techniques, whilst duplicate visits enable estimations of error rates.

The fact that large samples are insensitive to random error is not particularly surprising [9-11]. Nevertheless it is worth re-stating this fact within the context of DSS field operations where the money and time currently spent on endlessly correcting DSS datasets with diminishing return as the 100% accurate dataset is approached, would perhaps be better spent on increasing the size of surveillance populations and the geographic spread of DSSs, or indeed on analysing the data and disseminating findings.

## Conclusion

The random introduction of errors and missing data in key parameters in a large 'gold standard' dataset had little noticeable affect on population and mortality profiles, demonstrating a high level of robustness of DSS data and tolerable margins of error that may exceed 20%. This observation should not be taken as justifying poor quality data, or sloppy quality control procedures. However, the expense and practicality of detecting and correcting random errors must be considered in relation to the benefits of such efforts and the intended use of the data. Overall, this simple investigation suggests that stakeholders in DSS studies, as well as regional, national and global policy makers, should use DSSs data with confidence.

## Abbreviations

DSS – Demographic Surveillance Site; BRHP – Butajira Rural Health Programme; Indepth – International Network of field sites with continuous Demographic Evaluation of Populations and Their Health; NCD – Non-Communicable Disease; HIV/AIDS – Human Immunodeficiency Virus/Acquired Immunodeficiency Syndrome.

## Competing interests

The author(s) declare that they have no competing interests.

## Authors' contributions

EF: interpretation, drafting and revising manuscript; PB: design, data manipulation and revision of manuscript; YB: original data acquisition and supervision of BRHP activities, as well as interpretation of findings and manuscript revision. All authors read and approved the final manuscript.

## Pre-publication history

The pre-publication history for this paper can be accessed here:



## References

[B1] Beaglehole R, Bonita R (2001). Challenges for public health in the global context- prevention and surveillance.. Scandinavian Journal of Public Health.

[B2] Bonita R, Armstrong T Surveillance of Non-communicable disease risk factors.. http://www.who.int/mediacentre/factsheets/2003/fs273/en/.

[B3] Byass P (2001). Person, place and time- but who, where, and when?. Scandinavian Journal of Public Health.

[B4] Byass P, Berhane Y, Emmelin A, Kebede D, Andersson T, Hoberg U, Wall S (2002). The role of demographic surveillance systems (DSS) in assessing the health of communities: an example from rural Ethiopia.. Public Health.

[B5] Indepth Founding Document. http://www.indepth-network.org/core_documents/constituting_document_11_10_98.htm.

[B6] Indepth (2002). Population and Health in Developing Countries, Volume 1: Population, Health and Survival..

[B7] Setel PW, Sankoh O, Rao C, Velkoff VA, Mathers C, Gonghuan Y, Hemed Y, Jha P, Lopez AD (2006). Sample registration of vital events with verbal autopsy: a renewed commitment to measuring and monitoring vital statistics. Bulletin of the World Health Organisation.

[B8] Kirkwood BR, Sterne JAC (2003). Essential Medical Statistics, 2nd Edition.

[B9] Armstrong BG (1998). Effect of measurement error on epidemiological studies and occupational exposures. Occupational and Environmental Medicine.

[B10] Wong MY, Day NE, Bashir SA, Duffy SW (1999). Measurement error in epidemiology: the design of validation studies I: univariate situation. Statistics in Medicine.

[B11] Wong MY, Day NE, Wareham NJ (1999). Measurement error in epidemiology: the design of validation studies II: bivariate situation. Statistics in Medicine.

[B12] White I, Frost C, Tokunaga S (2001). Correcting for measurement error in binary and continuous variables using replicates. Statistics in Medicine.

[B13] White IR (2006). Dealing with measurement error: multiple imputation or regression calibration?. International Journal of Epidemiology.

[B14] Cole S, Chu, Greenland S (2006). Multiple-imputation for measurement error correction. International Journal of Epidemiology.

[B15] Espeland MA, Hui SL (1987). A general approach to analysing epidemiologic data that contain misclasification errors. Biometrics.

[B16] Bashir SA, Duffy SW (1997). The correction of risk estimates for measurement error. Annals of Epidemiology.

[B17] Spiegelman D, Schneeweiss S, McDermott A (1997). Measurement error correction for logistic regression models with an "alloyed gold standard".. American Journal of Epidemiology.

[B18] Jurek A, Maldonado G, Church T, Greenland S (2004). Exposure measurement error is frequently ignored when interpreting epidemiologic study results. American Journal of Epidemiology.

[B19] Indepth International Network of Field Sites With Continuous Demographic Evaluation of Populations and Their Health in Developing Countries. http://www.indepth-network.org.

[B20] Rowan KM, Byass P, Snow RW (1987). On-line tropical epidemiology - a case-study from The Gambia. Methods of Information in Medicine.

[B21] Omran A (1971). The Epidemiologic Transition: A theory of the epidemiology of population change.. Milbank Quarterly.

[B22] Ng N, Van Minh H, Tesfaye F, Bonita R, Byass P, Stenland H, Weinehall L, Wall S (2006). Combining risk factors and demographic surveillance: potentials of WHO STEPS and INDEPTH methodologies for assessing epidemiological transition. Scandinavian Journal of Public Health.

[B23] Khan K, Garenne ML, Collinson MA, Tollman SM (2007). Mortality trends in a new South Africa: hard to make a fresh start. Scandinavian Journal of Public Health.

[B24] Ng N, Stenland H, Bonita R, Hakimi M, Wall S, Weinehall L (2006). Preventable risk factors for non-communicable diseases in rural Indonesia: prevalence study using WHO STEPS approach. Bulletin of the World Health Organisation.

